# Cyclization of Two Antimicrobial Peptides Improves Their Activity

**DOI:** 10.1021/acsomega.4c11466

**Published:** 2025-02-26

**Authors:** Saheli Mitra, Mei-Tung Chen, Francisca Stedman, Jedidiah Hernandez, Grace Kumble, Xi Kang, Churan Zhang, Grace Tang, Iris Reed, Ian Q. Daugherty, Wanqing Liu, Kevin Raphael Klucznik, Jeremy L. Ocloo, Alexander Anzhi Li, Jessie Klousnitzer, Frank Heinrich, Berthony Deslouches, Stephanie Tristram-Nagle

**Affiliations:** † Biological Physics Group, Physics Department, Carnegie Mellon University, Pittsburgh, Pennsylvania 15213, United States; ‡ Center for Neutron Research, National Institute of Standards and Technology, Gaithersburg, Maryland 20899, United States; § Department of Environmental and Occupational Health, University of Pittsburgh, Pittsburgh, Pennsylvania 15261, United States

## Abstract

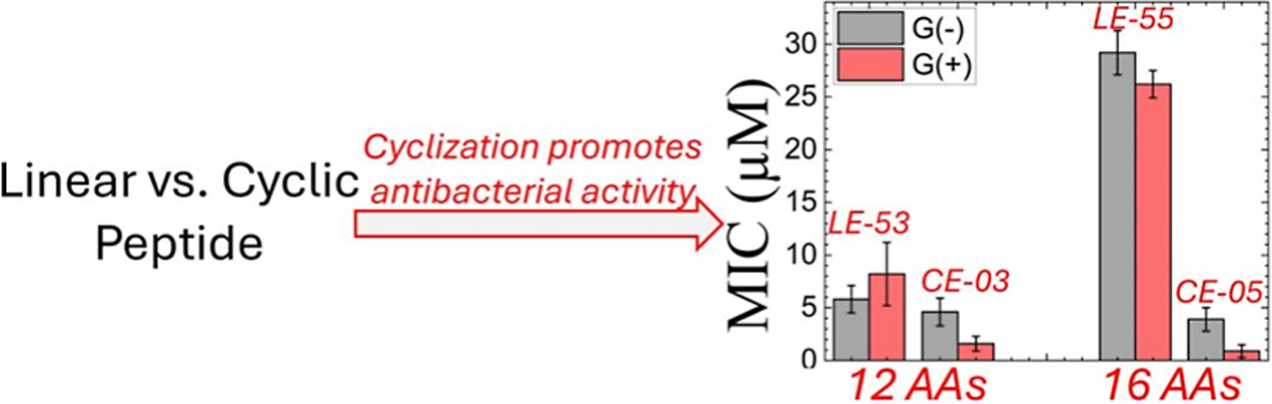

One promising strategy to combat worldwide antimicrobial resistance involves using cyclic peptides as antibacterial agents. Cyclization of peptides can confer several advantages, including enhanced stability to proteolysis, decreased toxicity and increased bactericidal efficacy. This paper examines two cyclic peptides CE-03 (12 AAs) and CE-05 (16 AAs) and evaluates their effectiveness in combating bacterial infections, their stability and toxicity. We compare them to their linear versions. Circular dichroism (CD) reveals that CE-03 and CE-05 both adopt random coil and β-sheet structures in lipid model membranes (LMMs) mimicking G(−) and G(+) bacteria, where they are both bactericidal. Using X-ray diffuse scattering (XDS), their effects on lipid model membranes show a deep penetration of both peptides into eukaryotic LMMs where they are nontoxic, while a headgroup location in bacterial LMMs correlates with bacterial killing. Neutron reflectometry (NR) confirms the AMP locations determined using XDS. Further, solution small-angle X-ray scattering demonstrates that both peptides induce vesicle fusion in bacterial LMMs without affecting eukaryotic LMMs. Proteolytic degradation studies show that both CE-05 and CE-03 do not lose activity when incubated with the elastase enzyme, while the helical E2-35 AMP becomes inactive upon proteolysis.

## Introduction

A 2023 report by the World Health Organization (WHO) states that antibacterial agents in the clinical pipeline combined with those approved in the last six years are still insufficient to tackle the emergence and spread of drug-resistant infections.^1^ In 2019 alone, antimicrobial resistance (AMR) was associated with the deaths of 4.95 million individuals worldwide.^2^ One especially deadly bacteria is the Gram-negative carbapenem-resistant *Acinetobacter baumannii* (CRAB), where mortality rates range between 40% and 60%, and are even higher in critically ill patients.^3^ The WHO reported that several new preclinical and clinical antibacterial agents are being developed; most are derivatives of traditional antibiotics, while a few are nontraditional agents. Two new nontraditional agents are membrane disruptors: cyclic peptides OMN6 (40 amino acids) and murepavadin (14 amino acids), which are both in early clinical trials.^1^ While OMN6 is nontoxic and stable toward proteolytic degradation, it targets only Gram-negative bacteria.^4^ Murepavadin is also selective in that it targets LptD, an outer membrane lipopolysaccharide protein transporter in Gram-negative *Pseudomonas aeruginosa*;^5^ it has also been shown to be nephrotoxic when delivered systemically.^6^ What is still needed is a broad-spectrum, nontoxic, proteolytically stable and nondrug-resistant antibacterial agent. This is the motivation for the current work.

Our lab has been inspired by the naturally occurring defense peptide, LL-37, which is a helical peptide containing 37 amino acids, including hydrophobic and cationic residues. Despite its strong antimicrobial properties, LL-37 has several limitations, including high cost, lower activity in physiological environments, susceptibility to proteolytic degradation and high toxicity to human cells.^7^ By limiting the length of antimicrobial peptides (AMPs) to 10–24 residues, the number of types of amino acids to 3, the incorporation of unnatural amino acids and the use of tryptophan (W) to ensure activity in physiological environments, our lab has pioneered several successful AMPs in preclinical development.^8−17^ One of these, WLBU2 (24-mer), is in Phase II clinical trials for infections related to knee arthroplasty. However, WLBU2 displays some toxicity in tests with red and white blood cells,^18^ so there is still room for improvement. Besides the variations mentioned above, other attempts to improve AMPs include stapling peptides to maintain an α-helical structure,^19^ and cyclizing a linear peptide. Both of these variations improve binding specificity and proteolytic stability.^20−22^ Drawing from nature, *Bacillus* bacterial species produce three main families of cyclic lipopeptides which contain a fatty acid attached to the cyclic peptide.^23^ The addition of a fatty acid to the peptide increases its permeation into the membrane, but this can also increase its toxicity to eukaryotic cells.^24^ By copying nature, cyclic peptoid polymers exert strong activity against drug-resistant bacteria.^25^

While many variations may be useful, the present work examines the secondary structure of two novel, synthetic peptides, CE-03 (12-mer) and CE-05 (16-mer), that are the cyclic forms of two linear amphipathic AMPs that we studied previously.^26^ Like their parent AMP WLBU2, these AMPs contain only three types of amino acids: valine (V), tryptophan (W) and arginine (R). We use circular dichroism (CD) to obtain their secondary structure, and X-ray diffuse scattering (XDS) to obtain the elastic properties and membrane structure of G(−) inner membrane (IM), G(+) and eukaryotic lipid model membranes (LMMs) when encountering the two cyclic AMPs. We obtain the location of the AMPs in the membrane, the perturbation in membrane thickness and the area per lipid of the membranes caused by the two peptides, and changes in rigidity and chain order of the LMMs. Neutron reflectometry (NR) experiments serve to validate the X-ray findings. Additionally, solution small-angle X-ray scattering was employed to investigate the fusogenic properties of these peptides. These biophysical results are combined with microbiological results in an effort to understand the mechanism of the membrane destabilization caused by these two cyclic AMPs. These structural and functional results for the cyclic AMPs are then compared to their linear counterparts.

## Results

### Physical Attributes and Activity

Table 1 shows the physical attributes of both CE-03 and CE-05. They are both highly cationic with 6 and 8 arginines, respectively. While the ratio of cationic to hydrophobic residues is 1 in each case, CE-03 has a higher hydrophobicity (H) as calculated by Heliquest^27^ due to the higher W/V ratio.

**Table 1:** Amino Acid Sequences of the Peptides and Their Physical Attributes^a^

peptide	peptide primary sequence	#AA	charge	H
CE-03	cyclo-(**RR RR RR** WW WW VV)	12	+6	0.448
CE-05	cyclo-(**RR RR RR RR** WW WW VV VV)	16	+8	0.362

a Charged residues are bolded. The structures of the linear peptides are embedded within the parentheses. See Materials and Methods for synthetic procedures.

Microbiological assays as described in Materials and Methods determine Minimum Inhibitory Concentration (MIC) values as a measure of the efficacy of each AMP at killing bacteria, where a lower MIC is more efficient. The average MIC values were lower for CE-03 and CE-05 compared to their linear counterparts (LE-53 and LE-55) for both G(−) and G(+) bacterial strains as shown in Table 2. This difference was particularly large for CE-05 compared to LE-05. A MIC average value of 29.2 μM for G(−) and 26.0 μM for G(+) are considered poor compared to values near 2–4 μM. Both cyclic peptides, like their linear counterparts, were found to be nontoxic to red blood cells (RBCs). Because 32 μM is such a high concentration compared to what would be a therapeutic dose, we consider anything less than 20% to be nontoxic.

**Table 2:** Antibacterial Activity and Toxicity of CE-03, LE-53, CE-05 and LE-55 Peptides^a^

antimicrobial	MIC (μM)									
peptide		G(−)					G(+)			% toxicity
	*PA*	*AB*	*KP*	*EC*	*Entbac*	average	*Entcoc*	*SA*	average	RBC
CE-03	7.1	2.2	8.2	1.5	3.7	4.6 ± 1.3	0.9	2.3	1.6 ± 0.7	4.2
LE-53	10.8	3.3	3.6	4.8	6.5	5.8 ± 1.3	14.4	2.0	8.2 ± 3.0	7.1
CE-05	6.1	1.1	6.7	2.5	3.2	3.9 ± 1.1	0.6	1.0	0.9 ± 0.6	14.5
LE-55	32.0	21.3	32.0	28.8	32.0	29.2 ± 2.1	28.0	24.0	26.0 ± 1.3	0.0
colistin	8.4	0.5	0.7	4.3	12.1	5.2 ± 4.3	32.0	64.0	48.0 ± 23.0	
tobramycin	32.0	32.0	2.1	28.0	24.5	23.7 ± 3.4	25.0	13.1	19.0 ± 1.0	

a Average MICs from five different species of G(−) bacteria were averaged for each AMP, and from two different species of G(+) bacteria. The average MICs for each bacterial species resulted from testing four strains. The G(−) bacterial strains are *Pseudomonas aerginosa* (*PA231*, *PA235*, *PA239*, *PA249*), *Acinetobacter baumannii* (*AB78*, *AB83*, *AB273*, *AB275*), *Klebsiella pneumoniae* (*KP106*, *KP506*, *KP542*, *KP550*), *Escherichia coli* (*EC541*, *EC543*, *EC546*, *EC549*) and *Enterobacter* (*EA62*, *EC544*, *EC547*, *EA1042*). The G(+) bacterial strains are *Enterococci* (*EF500*, *EF678*, *EF679*, *EF787*) and *Staphylococcus aureus* (*SA703*, *SA722*, *SA729*). Standard deviations of each of these averages were not shown so as not to overclutter the table. Values for LE-53 and LE-55 were taken from ref (26) with permission. % RBC lysis at 32 μM of AMP is shown.

### Proteolytic Degradation

One motivation in designing linear or cyclic AMPs is to prevent proteolytic degradation that can occur with helical peptides. In this work, proteolytic degradation was compared to E2-35, which we previously determined to be largely helical.^28^Table 3 shows the MIC values determined for AMPs preincubated with neutrophil elastase (NE) for 1 or 4 h or with ammonium bicarbonate (AB) as a control, before determining MIC values. Linezolid, a conventional antibiotic was included for comparison. MIC values remained similar to control for LE-53, CE-03 and CE-05 AMPs at 1 and 4 h of elastase degradation, indicating that these peptides are resistant to proteolysis. Surprisingly, the MIC value for LE-55 was lower after proteolytic digestion than in the control sample. E2-35 was degraded by proteolysis as shown by a lower efficacy, and linezolid had a higher MIC than the AMPs, except for the 4-Hr NE digestion of E2-35.

**Table 3:** Effect of Neutrophil Elastase Degradation on MIC^a^

AMP	1-Hr AB incubation	4-Hr AB incubation	1-Hr NE digestion	4-Hr NE digestion
LE-53	2.0 ± 0	3.0 ± 1.4	2.0 ± 0	1.5 ± 0.7
CE-03	1.0 ± 0.0	1.0 ± 0	1.5 ± 0.7	1.0 ± 0
LE-55	16.0 ± 0	6.0 ± 2.8	3.0 ± 1.4	3.0 ± 1.4
CE-05	6.0 ± 2.8	4.0 ± 0	3.0 ± 1.4	3.0 ± 1.4
E2–35	1.0 ± 0	1.0 ± 0	10.1 ± 8.5	>16 ± 0
linezolid	>16 ± 0	>16 ± 0	>16 ± 0	>16 ± 0

a MIC values (μmol/L) are shown for 1 h and 4 h incubations in ammonium bicarbonate (AB, control), and neutrophil elastase (NE). The bacteria used in the MIC assays is *Staphylococcus hemolyticus* 730 from the CDC antimicrobial resistance isolate bank.

### Secondary Structural Changes

Secondary structural changes are crucial to understanding protein folding as AMPs interact with bacterial and eukaryotic membranes. For bacterial membranes, we have chosen the inner membrane lipid composition for Gram-negative bacteria,^29^ even though AMPs first encounter lipopolysaccharide (LPS) on the bacterial outer membrane. The AMP must ultimately encounter the inner membrane to breach it, leading to bacterial killing. We chose an average composition for Gram-negative and Gram-positive membranes based on many bacterial strains.^29^ For the eukaryotic cell membrane we have selected a lipid composition that matches headgroup and chain composition in good approximation.^30^

**Figure 1:**
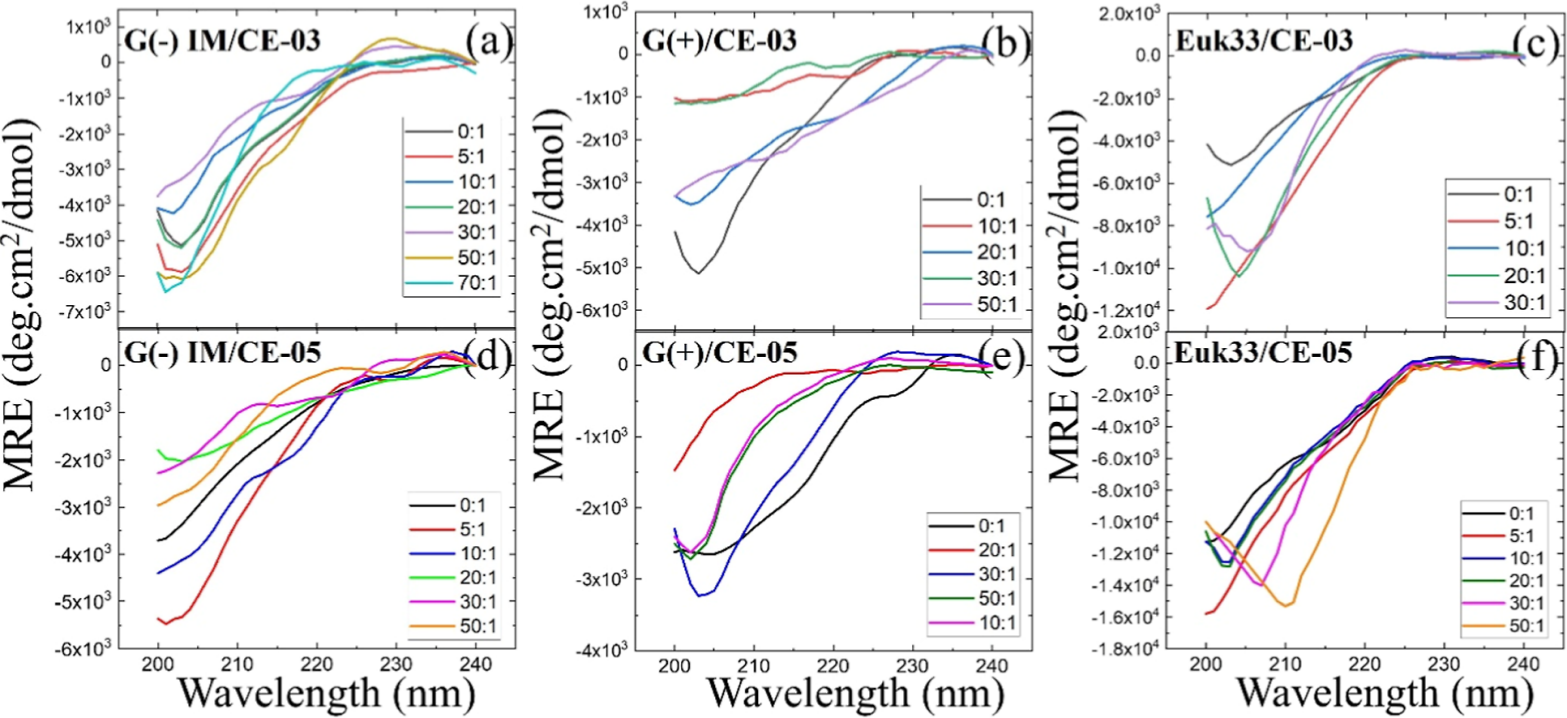
Mean residue ellipticity (MRE) as a function of lipid-to-peptide molar ratio. (a) G(−)/CE-03, (b) G(+)/CE-03, (c) Euk33/CE-03, (d) G(−)/CE-05, (e) G(+)/CE-05, (f) Euk33/CE-05. 0:1 (black lines) are pure peptides in 15 mmol/L phosphate buffer. Lipid is ULVs as described in Materials and Methods. Traces are smoothed using adjacent averaging (±5 nm).

**Figure 2:**
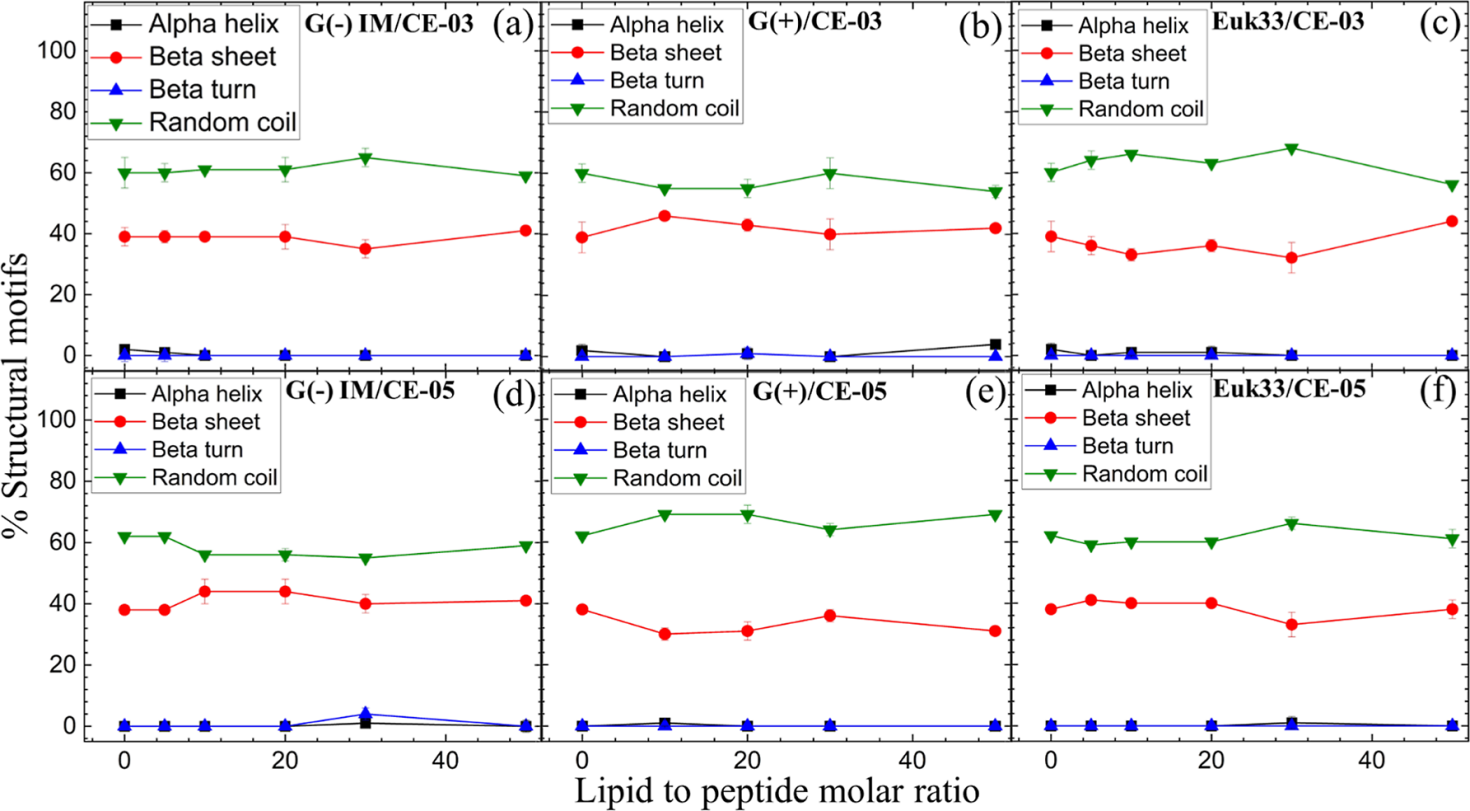
Percent structural motifs vs lipid/peptide molar ratio determined using CD spectroscopy. (a) G(−)/CE-03, (b) G(+)/CE03, (c) Euk33/CE-03, (d) G(−)/CE-05, (e) G(+)/CE-05, (f) Euk33/CE-05. IM indicates inner membrane of G(−) bacterial mimic. Error bars are 1 standard deviation.

As shown in Figure 1, there was no typical helical structure for pure peptide or peptide at any molar ratio with ULVs. Levenberg–Marquardt fitting to four structural motifs found nearly zero percent of α-helix or β-turn for any of the LMMs for either AMP. The major structural component is random coil at ∼60%, followed by β-sheet at ∼40%. These results are graphed in Figure 2. For CE-03 there is a small decrease in random coil and an increase in β-sheet at the highest lipid/peptide molar ratio (50:1). For CE-05, the trend is variable. See Tables S1–S6 in Supporting Information.

### Bending Moduli and Lipid Chain Order Parameters

**Figure 3:**
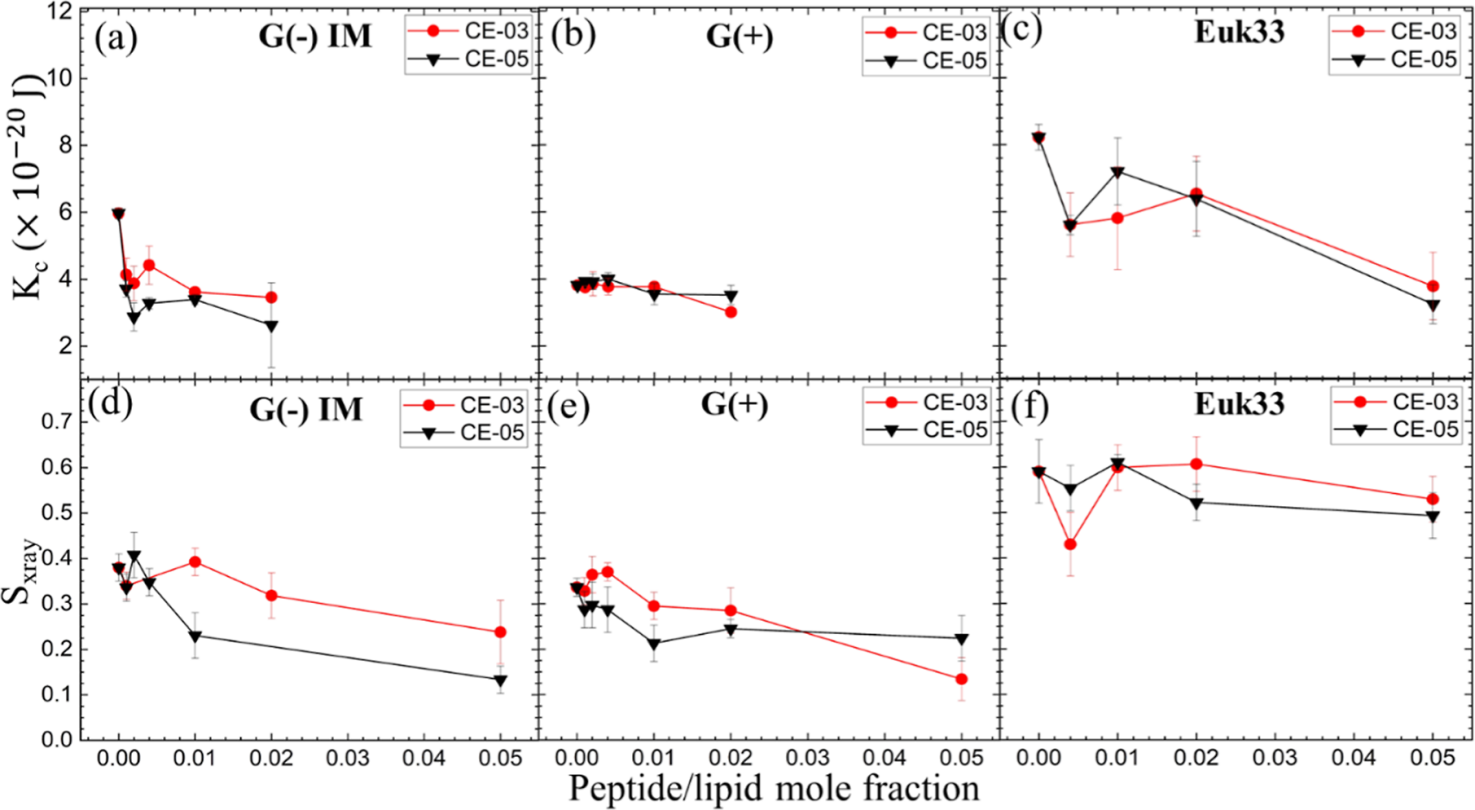
Bending modulus (*K*_C_) vs peptide/lipid mole fraction for (a) AMP/G(−), (b) AMP/G(+) and (c) AMP/Euk33. Chain order parameter (*S*_xray_) for (d) AMP/G(−), (e) AMP/G(+) and (f) AMP/Euk33. CE-03 (red solid circles), CE-05 (black solid triangles. Error bars are 1 standard deviation.

Herein, we collected X-ray diffuse scattering (XDS) to quantitate the change in membrane bending modulus (*K*_C_) and lipid chain order parameter (*S*_xray_) of LMMs with CE-03 and CE-05. Examples of the raw data used to obtain these moduli are shown in Figures S1 (LAXS) and S2 (WAXS). Figure 3a–c shows the elastic bending modulus (*K*_C_) of G(−)IM, G(+) and Euk33 LMMs with CE-03 and CE-05. A higher value of *K*_C_ indicates a stiffer membrane while a lower value indicates a softer membrane. A general softening was observed for both AMPs in G(−) and G(+) LMMs, and in Euk33 LMMs, suggesting that membrane softening is unrelated to either bacterial or eukaryotic toxicity.

In Figure 3d–f acyl chain order (*S*_xray_) is plotted vs peptide to lipid mole fraction. Higher values of *S*_xray_ signify ordered lipid acyl chains while lower values signify disordered lipid acyl chains. For lipid chain order, we observed only a slight disordering of chains in all three LMMs, suggesting that lipid chain order was also irrelevant to bacterial killing efficacy or toxicity.

### X-ray Structural Results

**Figure 4:**
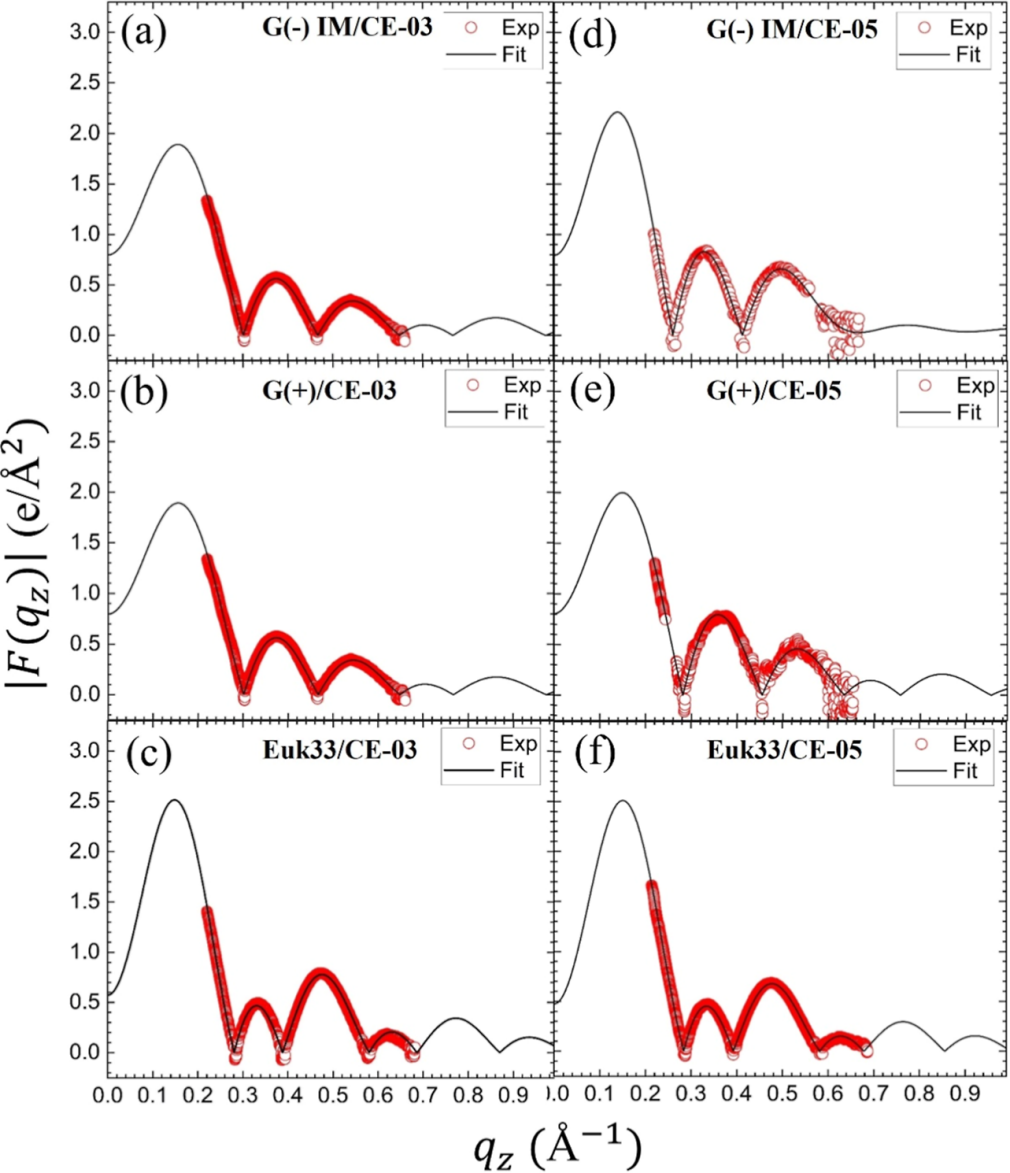
Form factors derived from LAXS data at lipid/peptide 50:1 molar ratio. Red open circles are experimental data points, and black lines are fits to the data using the scattering density profile (SDP) program. (a) G(−) inner membrane IM/CE-03 (b) G(+)/CE-03 (c) Euk33/CE-03 (d) G(−) IM/CE-05 (e) G(+)/CE-05 (f) Euk33/CE-05.

Figure 4 shows the form factors obtained from the liquid crystal theory fit to data as described in Materials and Methods. Each form factor is derived from the diffuse scattering intensity from a single, fully hydrated sample such as that shown in Figure S1. In addition to providing the bending modulus and *S*_xray_ order parameter, the same sample provides the intensity data along the entire *q-*range from 0.2 to 0.6 Å^–1^. The form factors are obtained by taking the square root of the intensity, and making the Lorentz and absorption corrections.^31^ They are related to the bilayer electron density profiles through the Fourier transform and model fitting.^32^

**Figure 5:**
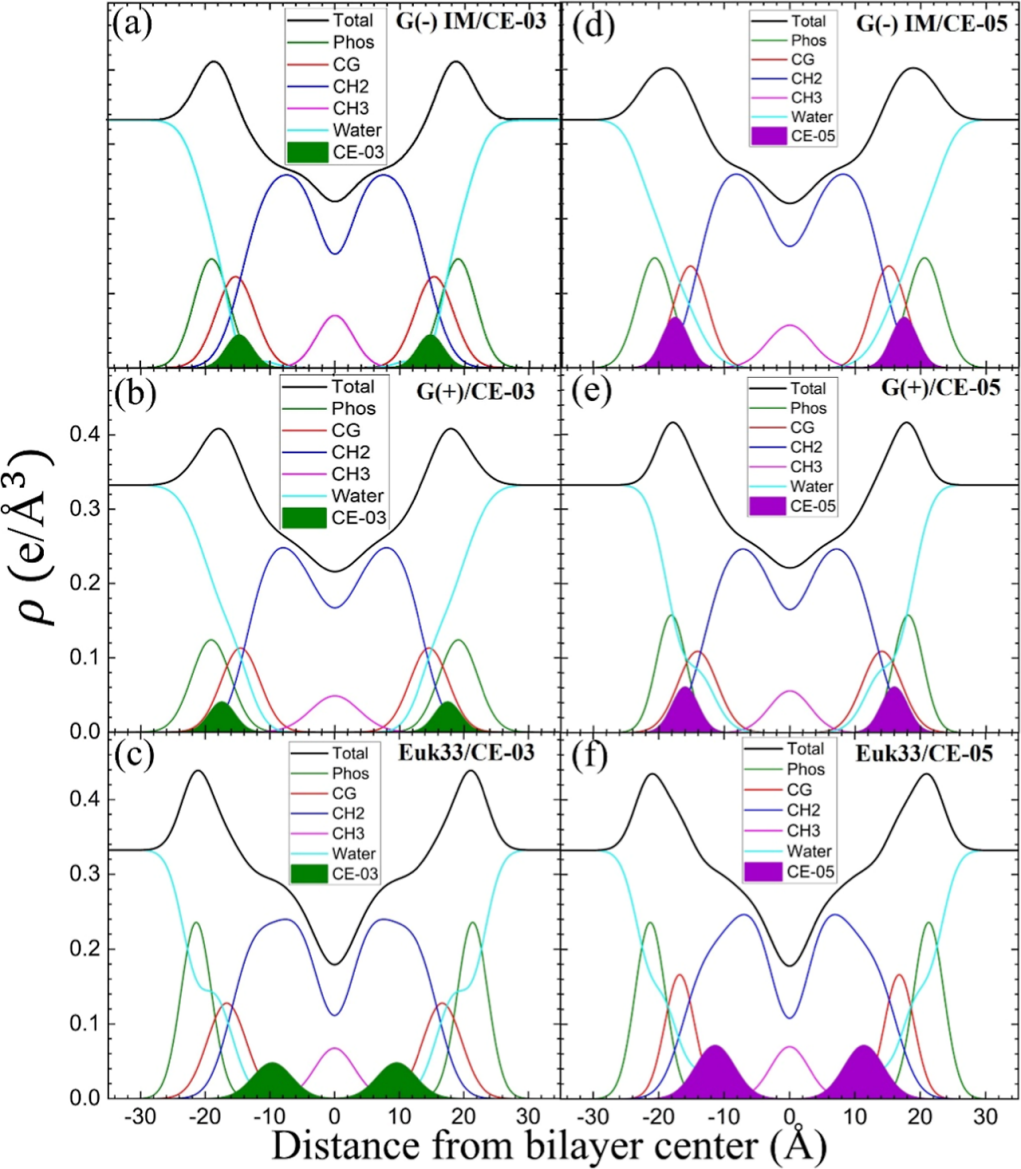
Electron density profiles obtained using the SDP program for the form factor data in Figure 4. Bilayer components are total (black), Phos (phosphate and external headgroup, green), CG (carbonyl-glycerol, red), CH2 (methylene hydrocarbon region, blue), CH_3_ (methyl trough, magenta), AMPs (CE-03, solid green, and CE-05, solid purple). Water (cyan) fills volumes around other groups to maintain a total probability of one. (a) G(−)IM/CE-03 (b) G(+)/CE-03 (c) Euk33/CE-03 (d) G(−) IM/CE-05 (e) G(+)/CE-05 (f) Euk33/CE-05.

With the scattering density profile (SDP) program^32^ we located the peptides in lipid bilayers, to attempt to make a correlation to bacterial killing efficacy. Figure 5 shows the electron density profiles (EDPs) obtained using SDP. SDP is based on the volumes and number of electrons of the different components listed in the figure legend of Figure 5. The volumes are fit to a bilayer model where the Gaussians and error functions are allowed to move along the bilayer depth (*z* direction). We place a Gaussian envelope for the peptide in three potential locations: the headgroup, hydrocarbon, or a combination of both, then assess the fit quality using the reduced chi-square metric. Key measures derived from these EDPs include the combined peak-to-peak distance (*D*_HH_) of phosphate and external headgroups (Phos) plus carbonyl-glycerol (CG), and the full-width at half-maximum of the envelope representing the hydrocarbon region (2D_C_), both of which indicate membrane thickness. The EDP also yields the area per lipid molecule (*A*_L_) using lipid and peptide volumes.

A summary of the XDS structural results from LAXS data for the three LMMs used in this study interacting with CE-03 and CE-05 is shown in Table 4. The addition of CE-03 and CE-05 to G(−) IM and G(+) LMMs caused an increase in A_L_ compared to the control, whereby this effect was more pronounced for the G(+) LMM. The increase in A_L_ was accompanied by a decrease in membrane thickness measured by D_HH_ and 2D_C_. For Euk33 LMMs, the opposite occurred; A_L_ decreased and the hydrocarbon membrane thickness increased when either AMP was added to the control.

**Table 4:** Summary of Structural Results from XDS

sample lipid/peptide = 50:1	area/lipid A_L_ [Å^2^] (±1.0)	D_HH_ [Å] (±0.5)	2D_C_ [Å] (±0.5)
**G(−) IM control**	71.4	39.8	29.0
G(−) IM/CE-03	72.8	37.4	28.4
G(−) IM/CE-05	73.4	37.9	28.1
**G(+) control**	72.5	37.2	29.3
G(+)/CE-03	78.9	35.9	26.9
G(+)/CE-05	81.7	35.8	25.9
**Euk33 control**	71.5	42.3	29.1
Euk33/CE-03	69.7	42.4	31.1
Euk33/CE-05	70.9	42.1	31.4

### Neutron Reflectivity Structural Results

**Figure 6:**
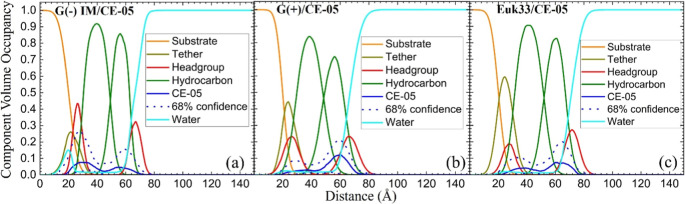
Neutron reflectivity component volume occupancy results for CE-05 interacting with G(−) (Figure 6a), G(+) (Figure 6b) and Euk33 (Figure 6c) LMMs. Component groups are shown in the legends.

Figure 6 shows the volume occupancy obtained using neutron reflectivity (NR) for CE-05 in LMMs. We use NR to confirm the location of the peptides in LMMs, since the scattering contrast between the peptide and the lipid bilayer and solvent is larger for neutrons than for X-rays. Data for CE-03 (not shown) were limited to G(+) and Euk33 LMMs due to difficulties in obtaining neutron beamtime. In G(−) (Figure 6a) and G(+) (Figure 6b) LMMs, CE-05 is located primarily in the headgroup region, similar to the X-ray results shown in Figure 5d,e. For Euk33 LMMs, CE-05 is primarily located in the hydrocarbon region, although also partially in the headgroup region (Figure 6c). NR results for CE-03 in G(+) and Euk33 LMMs were nearly identical to those for CE-05. Figure S3 shows the raw NR data that were used to calculate the Component Volume Occupancies shown in Figure 6. Table S7 quantitates the NR results.

**Figure 7:**
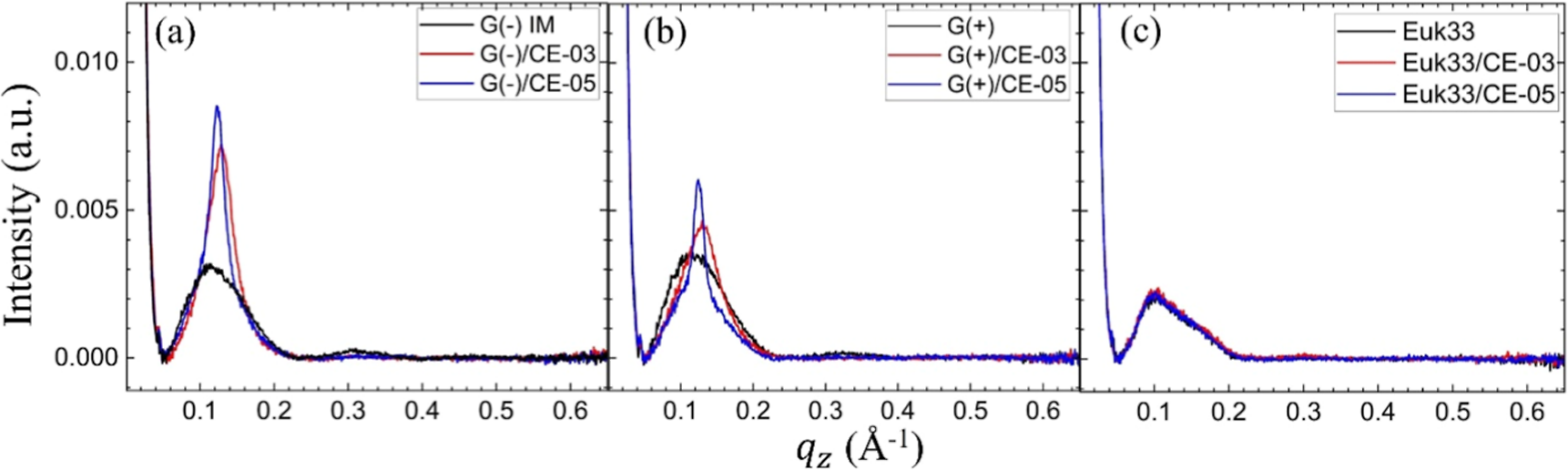
Intensity plots of small-angle X-ray scattering (SAXS) of ULVs with AMPs, 75:1 lipid/peptide molar ratio. (a) G(−) LMMs, (b) G(+) LMMs, (c) Euk33 LMMs. AMPs: CE-03 (red lines), CE-05 (blue lines) and control LMMs (black lines).

It was of interest to probe the fusogenicity of the AMPs when encountering ULVs of the three LMMs. As shown in Figure 7, both CE-03 and CE-05 caused the appearance of sharp peaks near *q* = 0.11 Å^–1^ due to Bragg lamellar orders that must have resulted from the formation of multilamellar vesicles (MLVs). This is evidence that the ULVs fused into larger structures with a discrete *D*-spacing between layers. The evidence for fusogenicity was strongest in G(−) LMMs, but also apparent in G(+) LMMs. In Euk33 LMMs, where the AMPs show no toxicity, no fusion occurred for either peptide.

## Discussion

**Figure 8:**
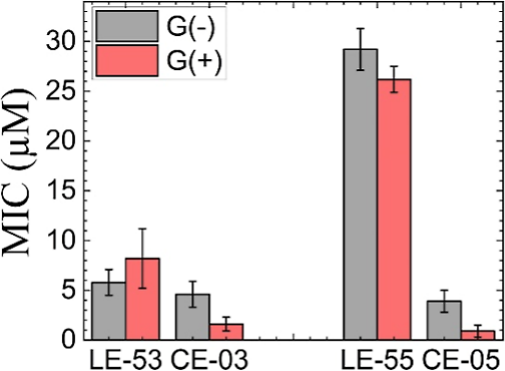
Comparison of MIC values of linear vs cyclic AMPs. G(−) filled gray, G(+) filled red. Cyclic AMP data shown are the averages from Table 2. Data for the LE peptides are adapted with permission from ref (26), 2024, the Royal Society of Chemistry. Error bars are 1 standard deviation.

This work compares two novel cyclic AMPs, CE-03 and CE-05, with their linear counterparts, which were recently published.^26^ CE-03 and LE-53 have 12 amino acids, while CE-05 and LE-55 have 16 amino acids. As summarized in Figure 8, both cyclic forms of these AMPs are more effective at killing bacteria than their linear forms. In the case of CE-05 compared to LE-55 there is a dramatic decrease in MIC due to cyclization. This was true for both G(−) and G(+) bacterial strains. For LE-53 a reduction in MIC was also observed due to cyclization, which was more significant in G(+) bacteria. Further, the cyclic peptides CE-03 and CE-05, as well as the linear peptide LE-53, demonstrated resistance to proteolytic degradation, maintaining similar MIC values after elastase digestion as in the control as shown in Table 3. In contrast, the helical peptide E2-35 showed reduced efficacy, highlighting the advantage of linear or cyclic peptides in resisting proteolysis. One interesting result was that proteolytic degradation actually increased the effectivity of LE-55. It is known that the enzyme elastase cleaves the peptide backbone at the carbonyl side of small hydrophobic amino acids, such as valine.^33^ This suggests that loss of the terminal valines was the reason for lowering the MIC. This result supports the idea that increased charge density and shorter peptides enhance AMP effectivity. This could also be the reason that cyclization enhances activity, since the cyclic peptides may have a higher charge density and are more compact than their linear forms.

The main question, then, is what is the biophysical source of this difference in activity? The CD results in Figures 1 and 2 show that both cyclical AMPs consist primarily of random coil and β-sheet, with almost no helical character. Yet, the same was true for the linear forms as we published previously.^26^ LE-53 is quite active at killing bacteria for the linear peptides, while LE-55 is inefficient.^26^ Together these results suggest that secondary structure is not a good predictor of bacterial killing efficiency. In a broader context, how is secondary structure related to bacterial killing efficacy? While two of our previous works demonstrated enhanced bactericidal efficacy with increased helicity of peptides in G(−) and G(+) LMMs,^28,34^ two of our other studies found excellent bacterial killing efficacy of nonhelical AMPs.^18,26^ These results were obtained via in vitro MIC assays, which may not always predict in vivo results. Many investigations have shown that secondary structures can vary dramatically among potent AMPs. α-Helical AMPs, such as magainin from the African clawed frog, are among the most intensely studied AMPs.^35,36^ Many other α-helical AMPs are effective at killing bacteria: Moricin, carnobacteriocin, novispirin, CA-MA and sheep myeloid antimicrobial peptide (see Table 1 in ref (37)). Alternatively, the β-sheet structure is present in many effective AMPs: Tachyplesin, β-defensin, lactoferricin, leucocin, protegrin, sapecin, androctonin, gomesin and heliomycin (see Table 1 in ref (37)). Other effective AMPs, such as indolicin, PW2 and tritrpticin exhibit extended or random conformations, while thanatin displays a loop conformation (see Table 1 in ref (37)). In addition, cyclic lipopeptides such as polymyxin B and E do not fit neatly into these structural categories. To answer the original question, there is no clear-cut relationship between helicity and bactericidal efficacy. Other investigations have considered the potency of cyclic AMPs compared to linear AMPs. A review article by Vogel et al.^38^ suggested that cyclization could enhance amphipathicity compared to linear AMPs, although the secondary structure is unaffected. This may offer some explanation for the stark contrast in efficacy (Figure 8) between CE-05 and LE-55.

The secondary structure of AMPs as they encounter membranes may correlate with their toxicity; it has long been thought that helical peptides are more toxic to eukaryotic cells than nonhelical peptides. Our group has found a positive correlation between helicity and toxicity for WLBU2,^18^ the E2-peptides,^28^ and SPLUNC1-derived antimicrobial peptides.^34^ We have also seen the converse, that nonhelical peptides are nontoxic,^18,26^ supporting the positive correlation between helicity and toxicity. Several literature studies have also found a correlation between α-helicity and toxicity to eukaryotic cells.^39−43^ In the present study, we found that neither cyclic peptide, CE-03 nor CE-05, is toxic to any measurable degree. Similarly, the linear forms, LE-53 and LE-55, were also nontoxic.^26^ Therefore, we have again found a correspondence between nonhelical structure and nontoxicity for all four peptides.

Material properties could offer some insight into the mechanisms of bacterial killing or toxicity. Previously we found a marked nonmonotonic behavior of the bending modulus (*K*_C_) when the AMP colistin (polymyxin E) interacted with G(−) LMMs, but not with G(+) or Euk33 LMMs.^44^ As such, there was a direct correlation with bacterial killing efficiency, since only G(−) bacteria are sensitive to colistin. We suggested that domain formation occurred as a function of increasing concentration of AMP in G(−) LMMs which could lead to weaknesses along the domain walls between rigid and soft domains, thus allowing water and ions to flow out of the bacteria. In the present work and in our published work on LE-53 and LE-55, we did not observe dramatic nonmonotonicity, just a general softening of all three LMMs, suggesting that nonmonotonic bending behavior (softening and stiffening) was not correlated with either bacterial or eukaryotic toxicity. Other investigations have attempted to use membrane mechanics to understand the energy costs of forming a pore. One approach to measure this energy cost is through line tension, which is defined as the energy per unit length required to maintain an edge, as in the hydrophilic–hydrophobic edge needed to form a pore or the edge between lipid phases in domain coexistence. May has suggested that if headgroup wrapping occurs around a pore (toroidal), then the line tension is 10 *k*_B_*T*/nm,^45^ implying an energetic cost of ∼100 *k*_B_*T* to create a 10 nm circumference pore.^46^ If an AMP binds to the headgroup region, this could reduce the line tension. One example of this is the cationic protegrin (PG-1), containing 16–18 amino acids, which causes worm-like projections observed by AFM.^47^ Molecular dynamics simulations supported this idea where monomers of PG-1 bind more strongly as the curvature of toroidal pores increases.^48^ As for the lipid chain region, we have observed gradual disordering of lipid chains for all three LMMs, suggesting that lipid chain disordering is not a major factor either in bacterial killing or toxicity in eukaryotic membranes.

The good fit of the SDP model to the experimental XDS form factor data in Figure 4 suggests that the structural results are accurate. In Table 4, the increase in area/lipid for G(−) and more so for G(+) LMMs as both AMPs are added is similar to our result with LE-53 and LE-55 (see Table 3 in ref (26)). The increase in area/lipid is accompanied by a decrease in membrane thickness which could facilitate the destabilization of the bacterial membranes. Interestingly these structural results were opposite for the eukaryotic membrane where the AMPs are nontoxic. Increasing the membrane thickness with a decrease in area/lipid could stabilize the eukaryotic membrane, thus preventing lysis. Standard deviations are obtained from multiple fittings of the same and different form factor data.

Figure 5 reveals the location of both AMPs in the three different LMMs. Both CE-03 and CE-05 lodge in the headgroup region in the bacterial LMMs, while LE-53 is located in the interfacial region and LE-55 is in the headgroup region.^26^ Concerning our previous discussion of line tension this headgroup location could be important for reducing the energy required for pore formation, but LE-55 is an outlier because it is less effective in killing bacteria. For eukaryotic membranes, we have previously found a correlation between a hydrocarbon peptide position and nontoxicity.^26,28^ XDS and NR confirm this for both the current linear^26^ and cyclic forms.

Finally our interesting finding that fusogencity of ULVs is correlated with the efficacy of bacterial killing for both linear and cyclic forms of these AMPs was unexpected. LE-55 was much less fusogenic than the other three AMPs,^26^ and is also less efficacious (Figure 8). During perturbation of the bacterial membrane by an AMP, fusion of bacterial membranes should not be required; in many instances a single bacterium will be attacked by one or more AMPs. Instead, we can think of the ability to fuse membranes as a measure of membrane destabilization. ULVs must fuse to form the lowest free-energy state of membranes, which is MLVs. This SAXS measurement is a probe-free, quick and easy test that we will use in the future to interrogate membrane destabilization by novel AMPs.

## Conclusions

This work used the biophysical techniques of CD, XDS, NR and SAXS to correlate membrane structure and properties with microbiological assays. While both cyclic AMPs are toxic to bacteria, they are nontoxic to eukaryotic cells. We found primarily random coil (∼60%) and β-sheet (∼40%) composition for the cyclic AMPs CE-03 and CE-05 in bacterial and eukaryotic LMMs, as was previously found for the linear forms, LE-53 and LE-55.^26^ Microbiological testing showed that the cyclic form (CE-05) of LE-55 is far superior at killing bacteria compared to LE-55, and that the cyclic form (CE-03) of LE-53 is slightly more efficacious than LE-53. Therefore, secondary structure and AMP efficacy are not correlated. Our material property results show a gradual softening caused by all four AMPs in all three LMMs, suggesting that bending modulus changes do not correlate with efficacy or toxicity. Lipid chain order also decreased somewhat for all the cyclic peptides as we have already reported for the parent linear peptides,^26^ suggesting that these changes also do not distinguish efficacy and toxicity. However, the X-ray structural results may be the most important biophysical results. The location of the AMPs in the bacterial LMMs is either in the headgroup or interfacial regions, which correlates with killing efficacy, perhaps by lowering the line tension needed for pore formation. For Euk33 LMMs, all four AMPs located in the hydrocarbon region, which could stabilize the membrane. The area per lipid increases in bacterial LMMs while the thickness decreases, which could destabilize membranes. For Euk33 LMMs, the area per lipid decreases and the membrane thickens, which could lead to stabilization. In addition, fusogenicity is correlated with bactericidal activity and nonfusogenicity is correlated with poor bactericidal activity and eukaryotic nontoxicity. Proteolytic studies showed that three AMPs (LE-53, CE-03 and CE-05) resist enzymatic degradation since they retained their bactericidal activity, even after 4 h of digestion. LE-55 even increased its efficacy after proteolysis, suggesting that loss of valines may increase activity, which could be due to an increase in charge density and compactness, similar to the effect of cyclization.

## Materials and Methods

### Materials

The synthetic lyophilized lipids 1-palmitoyl-2-oleoyl-*sn*-glycero-3-phosphoethanolamine (POPE), 1-palmitoyl-2-oleoyl-*sn*-glycero-3-phospho-(10-rac-glycerol) sodium salt (POPG), 10,30-bis[1,2-dioleoyl-*sn*-glycero-3-phospho]-*sn*-glycerol sodium salt (TOCL, i.e., cardiolipin), 1-stearoyl-2-oleoyl-*sn*-glycero-3-phosphocholine (SOPC), 1-palmitoyl-2-linoleoyl-*sn*-glycero-3-phosphocholine (PLPC), egg sphingomyelin (ESM), and 1,2-dioleoyl-3-trimeathylammoniumpropane chloride salt (DOTAP) were purchased from Avanti Polar Lipids (Alabaster, AL) and used as received. Cholesterol was from Nu-Chek-Prep (Waterville, MN). HPLC-grade organic solvents were purchased from Sigma-Aldrich (St. Louis, MO). Lipid stock solutions in chloroform were combined to create lipid mixtures in molar ratios mimicking the G(−) inner membrane (IM): POPE/POPG/TOCL (7:2:1 molar ratio), G(+) membrane: POPG/DOTAP/POPE/TOCL (6:1.5:1.5:1),^49^ and eukaryotic membrane, Euk33: SOPC/PLPC/POPE/ESM/cholesterol (15:10:5:3:16.5) (33 mol % cholesterol).^50^

Bacterial cation-adjusted Mueller Hinton Broth (MHB2), Test Condition Media, Roswell Park Memorial Institute (RPMI) media, fetal bovine serum (FBS) and phosphate-buffered saline (PBS) were obtained from Millipore Sigma (St Louis, MO). RPMI media contains the reducing agent glutathione as well as biotin, vitamin B12, and para aminobenzoic acid. In addition, RPMI media includes high concentrations of the vitamins inositol and choline. Because RPMI contains no proteins, lipids, or growth factors, it is commonly supplemented with FBS. FBS contains more than 1000 components such as growth factors, hormones, and transport proteins that contribute to cell growth when supplemented into culture media.^51^ Formaldehyde was obtained from ThermoFisher (Waltham, MA). Peptides were purchased in lyophilized form (10 mg in a 1.5 mL vial) from Genscript (Piscataway, NJ) with HPLC/MS spectra corresponding to each designed primary sequence. The traditional antibiotics and colistin were purchased from Millipore Sigma (St. Louis, MO). Amino acid sequences of the peptides and their physical attributes are provided in Table 1. For proteolysis studies, human neutrophil elastase was purchased from EMD Millipore Corporation, Burlington, MA.

### Methods

#### Peptide Synthesis

Solid phase peptide syntheses of CE-03 and CE-05 peptides were carried out at the University of Pittsburgh Peptide and Peptoid Synthesis Core accomplished on a Liberty CEM microwave synthesizer using Fmoc/*t*Bu chemistry and Oxyma pure coupling protocols on Wang resin solid supports. The completed linear peptide sequence containing resins were then cleaved with Trifluoroacetic acid (TFA) + scavengers followed by isolation of the crude products by precipitation in ice cold Diethyl Ether (EtO2). Crude linear peptides were then dissolved in 50%TFE/0.1% TFA and purified by preparative C-18 RP-HPLC on a Waters Delta Prep 4000 chromatography system followed by lyophilization to a dry powder. The purified linear peptides were then head-to-tail cyclized by dissolving to a concentration of 0.5 mg/mL in neat DMSO containing 60 mol of EDC (1-ethyl-3-(3-(dimethylamino)propyl) carbodiimide) and 20 mol of HOBt (*N*-hydroxbenzotriazole) per mole of peptide. Progress of the cyclization reactions were followed using C-18 RP-HPLC on an Alliance chromatography system using standard 0.1% TFA/Acetonitrile gradient conditions. The final peptide cyclization reaction solutions were then diluted 10-fold in ice cold 0.1%TFA and purified by preparative C-18 RP-HPLC on a Waters Delta Prep 4000 chromatography system followed by lyophilization to a dry powder. Confirmation of the correct theoretical mass of each peptide was verified on an Applied Biosystems TOF mass spectrometer using CHCA matrix conditions. HPLC and MS traces are shown in Figures S4–S7 in Supporting Information.

#### Antibacterial Assay

Bacterial clinical isolates used for initial screening were anonymously provided by the clinical microbiology laboratory of the University of Pittsburgh Medical Center (UPMC). Bacteria were stored at −80 °C and typically retrieved by obtaining single colonies on agar plates prior to subsequent liquid broth culture. Suspensions of test bacteria were prepared from the log phase of growth by diluting overnight cultures at 1:100 with fresh cation-adjusted Mueller–Hinton Broth (MHB2) and incubating for an additional 3–4 h. Bacteria were spun at 3000 *g* for 10 min. The pellet was resuspended in Test Condition Media to determine bacterial turbidity using a Den-1B densitometer (Grant Instruments, Beaver Falls, PA) at 0.5 McFarland units corresponding to 10^8^ CFU/mL.

To examine antibacterial activity, we used minor modifications of a standard growth inhibition assay endorsed by the Clinical and Laboratory Standards Institute (CLSI), as previously described.^13^ Bacteria were incubated with each of the indicated peptides in MHB2. The bacterial cells were kept in an incubator for 18 h at 37 °C, which is linked to a robotic system that feeds a plate reader every hour with one of 8 × 96-well plates. The 96-well plates are standard flat-bottom microliter plates purchased from Thermo Fisher (Waltham, MA). This setup allows the collection of growth kinetic data at A 570 (absorbance at 570 nm) to examine growth inhibition in real-time (BioTek Instruments, Winooski, VT). We define minimum inhibitory concentration (MIC) as the minimum peptide concentration that completely prevents bacterial growth, demonstrated by a flat (horizontal line) growth curve as a function of hourly determinations for 18 h at A570.^13,52^ The assays are typically repeated a second time. If the MIC differs from the first assay, a third experimental trial is performed to confirm the MIC.

#### Determination of Toxicity to Mammalian Cells

Toxicity to eukaryotic cells was examined using human red blood cells (RBCs).^52,53^ Briefly, RBCs were separated by histopaque differential centrifugation using blood anonymously obtained from the Central Blood Bank (Pittsburgh, PA). For the RBC lysis assay, the isolated RBCs were resuspended in PBS at a concentration of 5%. The peptides were serially diluted twofold in 100 μL of PBS before adding 100 μL of 5% RBC to a final dilution of 2.5% RBC to ensure that the A570 of hemoglobin did not saturate the plate reader. In parallel, the RBCs were osmotically burst with water at increasing concentrations to generate a standard curve of RBC lysis. Three technicians independently conducted experiments to ensure reproducibility.

#### Proteolytic Degradation

The neutrophil elastase was dissolved in 200 mmol/L Tris buffer, pH 8.8, and used at a molar ratio of 1:50 with the peptide in 200 mM ammonium bicarbonate pH 8.0 for 1 or 4 h. The control experiment incubated the peptides alone in 200 mmol/L ammonium bicarbonate for 1 or 4 h. Upon completion of the incubations, the peptides were serially diluted to test MIC. 50% (v/v) MHB2 was added to the plates. MIC was determined as described above.

#### Circular Dichroism (CD)

Unilamellar vesicles (ULVs) of ∼600 Å diameter were prepared using an extruder (Avanti Polar Lipids, Alabaster, AL). 250 μL of 20 mg/mL multilamellar lipid vesicles was extruded 25 times through a single Nucleopore filter of size 500 Å using 0.2 mL Hamilton syringes. The final lipid concentration in the ULVs was 18 mg/mL as determined gravimetrically. Concentrated ULVs were added to 3 mL of 10 μmol/L (μM) peptide in 15 mmol/L PBS at pH 7 to create lipid/peptide molar ratios between 0:1 and 50:1. Higher molar ratios of lipid/peptide were not possible due to absorption flattening in the UV region. The samples remained at room temperature for ∼1–4 h before the CD measurement. Data were collected in 3 mL quartz cuvettes using a JASCO 1500 CD spectrometer at 37 °C in the Chemistry Department at Carnegie Mellon University. The samples were scanned from 200 to 240 nm 20 times and the results averaged. The temperature was controlled at 37 °C via a Peltier element with water circulation through the sample compartment. Nitrogen gas was used at a flow rate between 0.56 and 0.71 m^3^/h to protect the UV bulb. OriginPro 2024 (OriginLab, Northampton, MA) was used to carry out a Levenberg–Marquardt least-squares fit of the tryptophan-subtracted ellipticity traces to four secondary structural motifs representing α-helix, β-sheet, β-turn and random coil.^18,54^ This analysis gives a percentage match of each secondary structural motif to the total sample ellipticity. Instrument ellipticity (ε) was converted to Mean Residue Ellipticity using MRE (deg cm^2^/dmol) = ε × 10^4^/*N*, where *N* = # amino acids and peptide concentration was always 10 μM.

#### Low-Angle X-ray Diffuse Scattering (LAXS, XDS)

Oriented samples consisting of stacks of approximately ∼1800 bilayers were prepared using the well-established “rock and roll” method.^55^ 4 mg of lipids and peptides in organic solvent, chloroform/methanol (2:1, v/v) or trifluoroethanol/chloroform (1:1, v/v), were deposited onto a Si wafer (15 mm W × 30 mm L × 1 mm H) inside a fume hood. After rapid evaporation while rocking the substrate, an immobile film formed which was then further dried inside the fume hood for 2 h, followed by overnight drying under vacuum to evaporate residual organic solvent. The samples were trimmed to occupy 5 mm W × 30 mm L along the center of the Si substrate. The substrate was fixed to a glass block (10 mm H × 15 mm W × 32 mm L) using heat sink compound (Dow Corning, Freeland, MI). The samples were stored in a refrigerator at 4 °C. Cold storage immediately prior to transfer into a well-insulated hydration chamber held at 37 °C caused 100% hydration through the vapor within just 10 min. This process is faster than our previous method that required a Peltier cooler under the sample.^56^ Low-angle XDS (LAXS) data from oriented, fully hydrated samples were obtained at the ID7B2 line at Center for High Energy X-ray Sciences (CHEXS, Ithaca, NY) on two separate trips to the Cornell High Energy Synchrotron Source (CHESS) using X-ray wavelengths of 0.8855 and 0.8856 Å, sample-to-detector (S)-distances of 401 and 400.1 mm, beam size 0.25 mm H and 0.35 mm V, with an Eiger 16 M detector. 30 s exposures were carried out in the fluid phase at 37 °C. The flat silicon wafer was rotated from −1 to 6° during the data collection at CHESS to equally sample all angles of incidence. The background was collected by setting the X-ray angle of incidence to −2.0°, where sample scattering does not contribute to the image. For data analysis, backgrounds were first subtracted to remove extraneous air and mylar scattering and the images were laterally symmetrized to increase the signal-to-noise ratio. As the sample nears full hydration, membrane fluctuations occur which produce “‘lobes’” of diffuse X-ray scattering data.^31^ The fluctuations are quantitated by measuring the falloff in lobe intensity in the lateral *q*_r_ direction. The fitting procedure is a nonlinear least-squares fit that uses the free energy functional from liquid crystal theory^57^
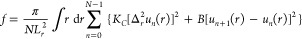
1where *N* is the number of bilayers in the vertical (*Z*) direction, *L*_r_ is the domain size in the horizontal (r) direction, and *K*_C_ is the bending modulus. *K*_C_ describes the bending of an average, single bilayer where u_n_ is the vertical membrane displacement and B is the compressibility modulus. A higher *K*_C_ indicates a stiffer membrane, and a lower *K*_C_ indicates a softer membrane.

#### Wide-angle X-ray Diffuse Scattering (WAXS, XDS)

Wide-angle XDS (WAXS) was obtained at CHESS. In order to obtain WAXS data, the same sample that was hydrated for LAXS is X-rayed with a fixed glancing angle of incidence, instead of a rotation of the sample. In order to remove significant water scattering in the wide-angle region, a gentle nitrogen stream was introduced into the hydration chamber during continuous WAXS data collection. Two exposures are taken at angles of X-ray incidence α = +0.3 and α = −0.3°, where the negative angle image is then subtracted from the positive angle image. Both are 30 s scans. The subtraction procedure removes extraneous scatter due to the mylar chamber windows and shadows. Excess water that condensed into the sample is removed by subtracting a water background formed on a clean silicon wafer; water is first scaled before subtraction for different water content in the samples. The chain–chain correlation appears as strong diffuse scatter projecting upward circularly from the equator; the falloff in azimuthal intensity yields information about chain order. To obtain an *S*_xray_ order parameter the subtracted intensity is first integrated along its radial trajectory, then fit to wide-angle liquid crystal theory.^58^ The chain scattering model assumes long thin rods that are locally well aligned along the local director (*n*_L_), with orientation described by the angle β. While acyl chains from lipids in the fluid phase are not long cylinders, the model allows the cylinders to tilt (β) in a Mauer-Saupe distribution to approximate chain disorder. From the fit of the intensity data using a Matlab computer program,^59^ we obtain *S*_xray_ using eq 2
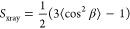
2

We also obtain the RMSE (root-mean-square error), which reports the goodness of the fit.

#### Neutron Reflectivity (NR)

NR measurements were performed at the OFFSPEC reflectometer^60^ at the ISIS Neutron and Muon Source, Rutherford Appleton Laboratory, Didcot, United Kingdom. Reflectivity curves were recorded at 37 °C for momentum transfer values 0.01 Å^–1^ ≤ *q*_*z*_ ≤ 0.25 Å^–1^. The neutron sample cells allowed in situ buffer exchange, and the same sample was incubated with H_2_O and D_2_O to provide scattering contrast.^61^ Six mg of lipid and peptide mixtures were cosolubilized in chloroform, dried under vacuum and rehydrated for 1–2 h via bath sonication in 1.2 mL 2 M NaCl, creating peptide-containing lipid vesicles. Sparsely tethered lipid bilayer membranes (stBLMs) were prepared on smooth gold-coated (∼140 Å film thickness, 4–9 Å r.m.s surface roughness) silicon wafers by immersing them in a 70:30 mol/mol β-mercaptoethanol/HC18 tether solution in ethanol for at least 60 min, leading to the formation of a self-assembled monolayer (SAM) of both molecules at the gold surface.^62^ SAM-decorated wafers were assembled in the NR cell, and lipid bilayers were completed by fusing vesicles of the desired lipid/peptide mixtures using an osmotic shock procedure. NR data were sequentially collected after rinsing the NR cell with ∼6 cell volumes of either D_2_O or H_2_O using a syringe. NR data sets collected on stBLMs immersed in isotopically different solutions were analyzed simultaneously (2 data sets per stBLM). One-dimensional structural profiles of the substrate and the lipid bilayer along the interface normal z were parametrized with a model that utilizes continuous volume occupancy distributions of the molecular components. Freeform peptide profiles were modeled using Hermite splines with control points on average 15 Å apart. A Monte Carlo Markov Chain-based global optimizer was used to determine best-fit parameters and their confidence limits, shown as 68% in the Component Volume Occupancy graph.

#### Solution Small Angle X-ray Scattering (SAXS) Measurements on ULVs

Solution SAXS measurements were performed on ULVs (prepared as described for CD spectrscopy) of lipids with embedded peptides using a Xeuss 3.0 (XENOCS, Holyoke, MA) instrument. The instrument features a Rigaku Cu Kα rotating anode source (λ ∼ 1.5418 Å) (The Woodlands, TX) and an Eiger 1 M detector (Dectris, Switzerland). The system was in the high-flux configuration with a scattering vector (q) range of 0.03 < *q* < 0.73 Å^–1^ with sample-to-detector distance = 370 mm. ULVs were robotically injected into the Xeuss BioCube flow cell to enable precise measurements of very small volumes (15 μL). Measurements were carried out at 37 °C with 600 s exposures. Scattering intensity (*I*) versus scattering vector *q* (*q* = 4π/λ sin(θ), where λ is the wavelength and 2θ is the scattering angle) was obtained by azimuthally averaging the 2D data. As demonstrated in ref (63), the absorption coefficient by ULV solution is independent of *q* over the range studied; hence, no absorption correction was required. Further, a linear intensity corresponding to pure water was subtracted from the acquired scattering intensity *I*(*q*).

## Supporting Information Available

Tables S1–S6, CD results of CE-03 and CE-05 in three LMMs; Figure S1, example of LAXS; Figure S2, example of WAXS; Figure S3, neutron reflectivity raw data; Table S7, quantitative results from composition-space fitting to NR scattering data; Figure S4, high-pressure liquid chromatograph (HPLC) of CE-03; Figure S5, mass spectrometry (MS) of CE-03; Figure S6, HPLC of CE-05; Figure S7, MS of CE-05 (PDF)
