# Supplementary material for: Cyclization of Two Antimicrobial Peptides Improves Their Activity
Source: ACS Omega. 2025 Feb 26;10(9):9728–40. doi: 10.1021/acsomega.4c11466 (PMC11904707; doi:10.1021/acsomega.4c11466)
Supplement: Supplementary file 1 — ao4c11466_si_001.pdf [file ao4c11466_si_001.pdf]

Supporting Information for Cyclization of two antimicrobial peptides improves their activity

Saheli Mitra[1], Mei-Tung Chen[1], Francisca Stedman[1], Jedidiah Hernandez[1], Grace Kumble[1], Xi Kang[1], Churan Zhang[1], Grace Tang[1], Iris Reed[1], Ian Q. Daugherty[1], Wanqing Liu[1], Kevin Raphael Klucznik[1], Jeremy L. Ocloo[1], Alexander Anzhi Li[1], Jessie Klousnitzer[3], Frank Heinrich[1,2], Berthony Deslouches[3*], Stephanie Tristram-Nagle[1*]

[1]Biological Physics Group, Physics Department, Carnegie Mellon University, Pittsburgh, PA 15213, USA; [2]Center for Neutron Research, National Institute of Standards and Technology, Gaithersburg, MD 20899, USA; [3]Department of Environmental and Occupational Health, University of Pittsburgh, Pittsburgh, PA 15261, USA

*Co-corresponding authors' emails: stn@cmu.edu, tdesl19@pitt.edu

Tables S1 – S6 summarize secondary structural results (% α-helix, β-sheet, β-turn and random coil) for CE-03 and CE-05 peptides in three LMMs. R^2 indicates the goodness of fit.

Table S1. CE-03 CD results of secondary structure in G(-) IM LMMs

G(-) IM/CE-03	α-helix (%)	β-sheet (%)	β-turn (%)	Random	Adj. R^2
0:1	2±2	39±5	0±0	60±3	0.98±0.02
5:1	1±2	39±3	0±0	60±2	0.96±0.01
10:1	0±0	39±1	0±0	61±1	0.99±0.01
20:1	0±0	39±4	0±0	61±4	0.96±0.04
30:1	0±0	35±3	0±0	65±3	0.95±0.04
50:1	0±0	41±1	0±0	59±1	0.98±0.03

Table S2. CE-03 CD results of secondary structure in G(+) LMMs

G(+) /E2-53R	α-helix (%)	β-sheet (%)	β-turn (%)	Random	Adj. R^2
0:1	2±2	39±5	0±0	60±3	0.98±0.02
10:1	0±0	46±0	0±0	55±0	0.81±0.02
20:1	1±2	43±2	1±2	55±3	0.93±0.09
30:1	0±0	40±5	0±0	60±5	0.95±0.01
50:1	4±0	42±0	0±0	54±2	0.92±0.01

Table S3. CE-03 CD results of secondary structure in Euk33 LMMs

Euk33 /CE-03	α-helix (%)	β-sheet (%)	β-turn (%)	Random	Adj, R^2
0:1	2±2	39±5	0±0	60±3	0.98±0.02
5:1	0±0	36±3	0±0	64±3	0.97±0.01
10:1	1±1	33±2	0±0	66±0	0.98±0.01
20:1	1±2	36±2	0±0	63±0	0.94±0.05
30:1	0±0	32±5	0±0	68±0	0.9±0.09
50:1	0±0	44±1	0±0	56±1	0.96±0.01

Table S4. CE-05 CD results of secondary structure in G(-) IM LMMs

G(-) IM/CE-05	α-helix	β-sheet (%)	β-turn (%)	Random	Adj. R^2
0:1	0±0	38±1	0±1	62±1	0.98±0.04
5:1	0±0	38±1	0±0	62±1	0.96±0.01
10:1	0±0	44±1	0±0	56±1	0.98±0.01
20:1	0±0	44±4	0±0	56±2	0.96±0.00
30:1	1±0	40±3	4±2	55±1	0.96±0.02
50:1	0±2	41±1	0±0	59±1	0.97±0.01

Table S5. CE-05 CD results of secondary structure in G(+) LMMs

G(+)/LE-54R	α-helix	β-sheet (%)	β-turn (%)	Random	Adj. R^2
0:1	0±0	38±1	0±1	62±1	0.98±0.04
10:1	1±1	30±2	0±0	69±1	0.98±0.02
20:1	0±0	31±3	0±0	69±3	0.95±0.01
30:1	0±0	36±2	0±0	64±2	0.92±0.06
50:1	0±0	31±1	0±0	69±1	0.99±0.01

Table S6. CE-05 CD results of secondary structure in Euk33 LMMs

Euk33/CE-05	α-helix	β-sheet (%)	β-turn (%)	Random	Adj. R^2
0:1	0±0	38±1	0±1	62±1	0.98±0.04
5:1	0±0	41±1	0±0	59±1	0.98±0.01
10:1	0±0	40±1	0±0	60±1	0.98±0.03
20:1	0±0	40±1	0±0	60±1	0.99±0.03
30:1	1±2	33±4	0±0	66±2	0.92±0.02
50:1	0±0	38±3	0±0	61±3	0.95±0.02

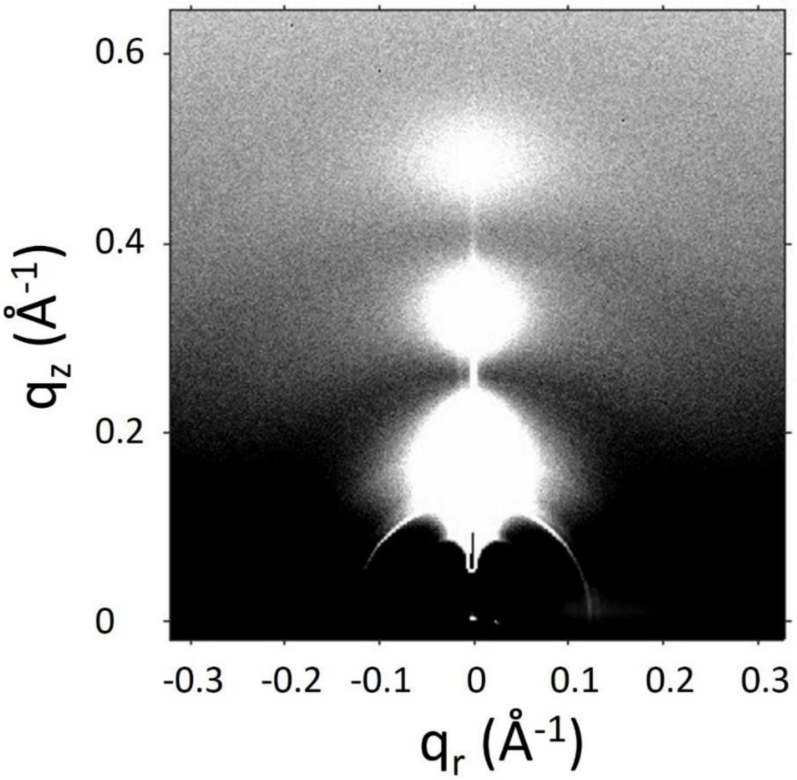

Figure S1. Low-angle x-ray scattering (LAXS) of G(-)/CE-05 (100:1) lipid:peptide molar ratio. A background scan collected at -2 degrees angle of incidence to the sample was subtracted.

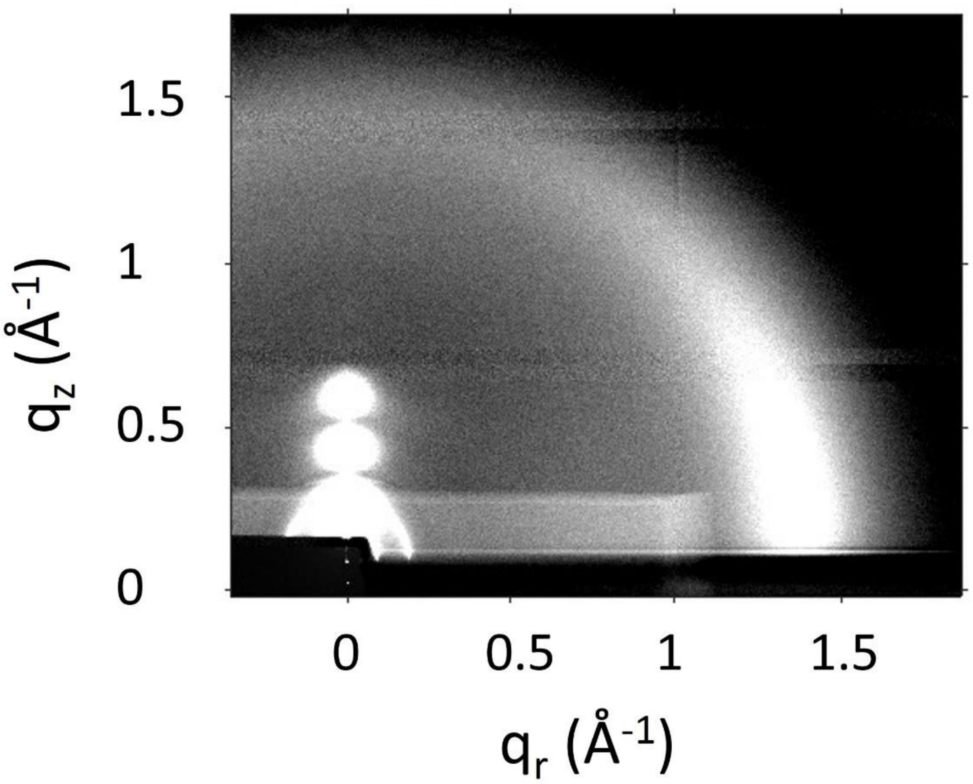

Figure S2. Wide-angle x-ray scattering (WAXS) of G(-)/CE-05 (1000:1) lipid:peptide molar ratio centered ~1.4 Å^{-1} in q_r. The foreground file was collected at 0.3 degrees angle of incidence. A background scan collected at -0.3 degrees was subtracted. A second background scan containing wide-angle scattering of water condensed onto a clean silicon wafer was also subtracted. The faint horizontal lines at ~0.7 and ~1.4 Å^{-1} in q_z result from attempts to erase the panel partitions in the Eiger 16M Dectris detector.

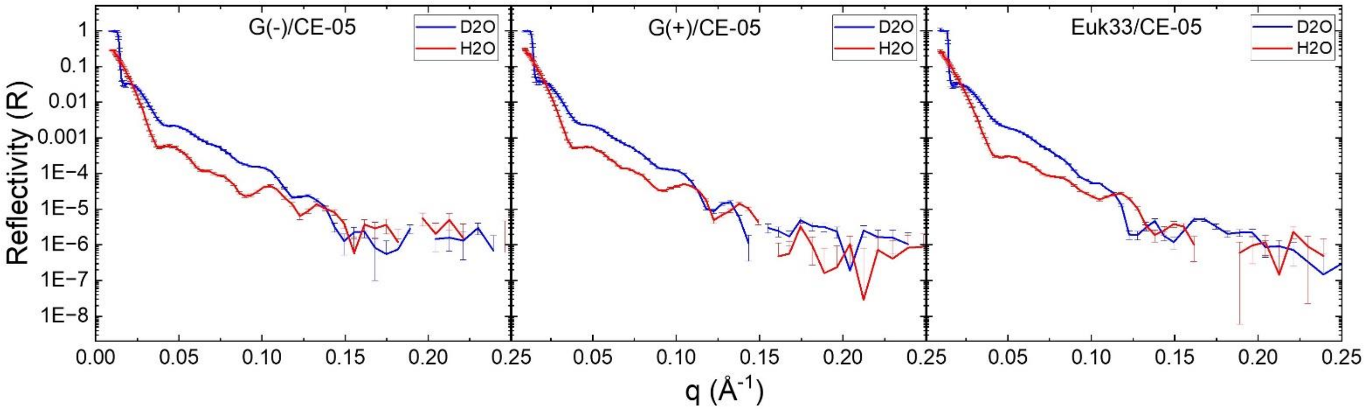

Figure S3. Neutron reflectivity raw data collected at ISIS at 37 ºC. Solvent is D_2O (blue traces) or H_2O (red traces).

Table S7. Quantitative results from Composition-Space Fitting to NR scattering data

**Parameter**	**G(-)IM/CE-05**	**G(+)/CE-05**	**Euk33/CE-05**
Substrate R.M.S. roughness / Å	5 ± 1	4 ± 1	4.0 ± 0.6
Bilayer R.M.S. roughness / Å	3.2 ± 0.9	4.8 ± 0.8	4.5 ± 0.8
Tether thickness / Å	9 ± 1	9 ± 1	11 ± 2
Hydrocarbon thickness inner lipid leaflet / Å	18 ± 3	18 ± 2	21 ± 3
Hydrocarbon thickness outer lipid leaflet / Å	14 ± 3	15 ± 3	15 ± 4
Area per lipid, outer leaflet / $Å^2$	80 ± 20	70 ± 10	58 ± 13
Bilayer completeness / %	98 ± 2	92 ± 2	98 ± 1
Peptide			
Amount of membrane-associated protein / $Å^3/Å^2$	4 ± 2	2± 1	3 ± 2
Fraction of protein sub-membrane	0.09±0.08	0.06±0.06	0.03±0.03
Fraction of protein in hydrocarbons	0.55±0.18	0.60±0.16	0.74±0.14
Fraction of protein in headgroups	0.27±0.08	0.26±0.08	0.22±0.12
Fraction of protein in bulk solvent	0.10±0.08	0.08±0.06	0.02±0.02
Peak position from headgroup / solvent interface / Å	-14±5	-13±7	-26 ± 15
Goodness of fit, chi-squared (D_2O, H_2O)	1.0, 1.4	3.9, 1.5	2.2

SAMPLE INFORMATION

Sample Name:	CE-0003 Cyclic, HPLC Pure,	Acquired By:	System
Sample Type:	Unknown	Sample Set Name:	
Vial:	1	Acq. Method Set:	ANALYTICAL
Injection #:	2	Processing Methoc	Luna C18_Analytical
Injection Volume:	25.00 ul	Channel Name:	W2489 ChA
Run Time:	45.0 Minutes	Proc. Chnl. Descr.:	W2489 ChA 220nm
Date Acquired:	11/28/2022 3:33:06 PM EST		
Date Processed:	11/29/2022 11:29:40 AM EST		

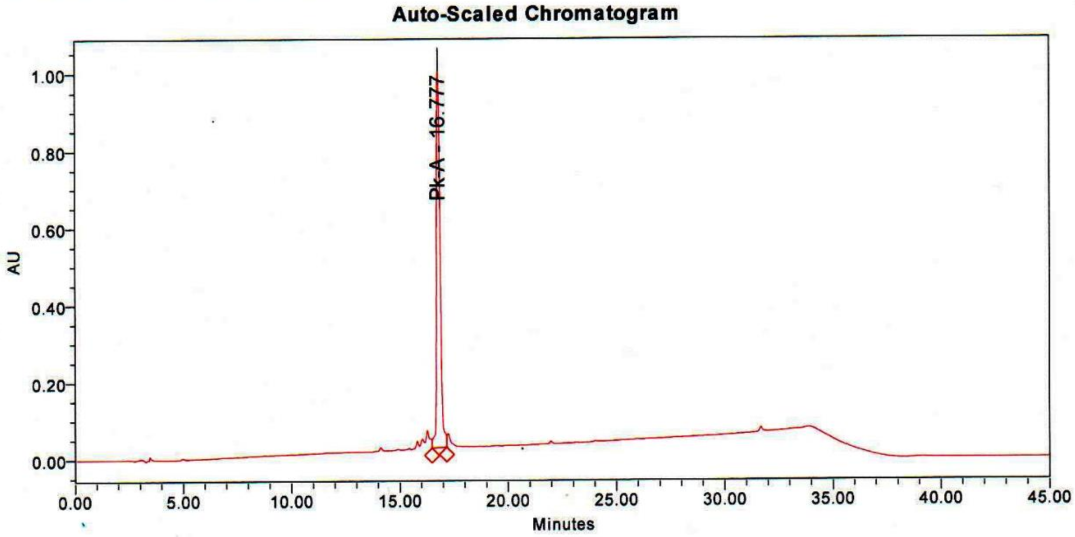

Column Information:
Waters Symmetry 5u C18 Analytical
UV Absorbance @ 220 nm
Injection: ~25ug: 0.1% TFA (aq.)
Mobile Phase:
A: 0.1% TFA (aq) B: 100% ACN / 0.1%

Time	Flow	%A	%B	Curve
Initial	1.0	95	5	*
35	1.0	30	70	6
40	1.0	95	5	6
50	0	95	5	11

Peak Results

	Name	RT	% Area
1	Pk-A	16.777	100.00

Figure S4. High-pressure liquid chromatograph of CE-03.

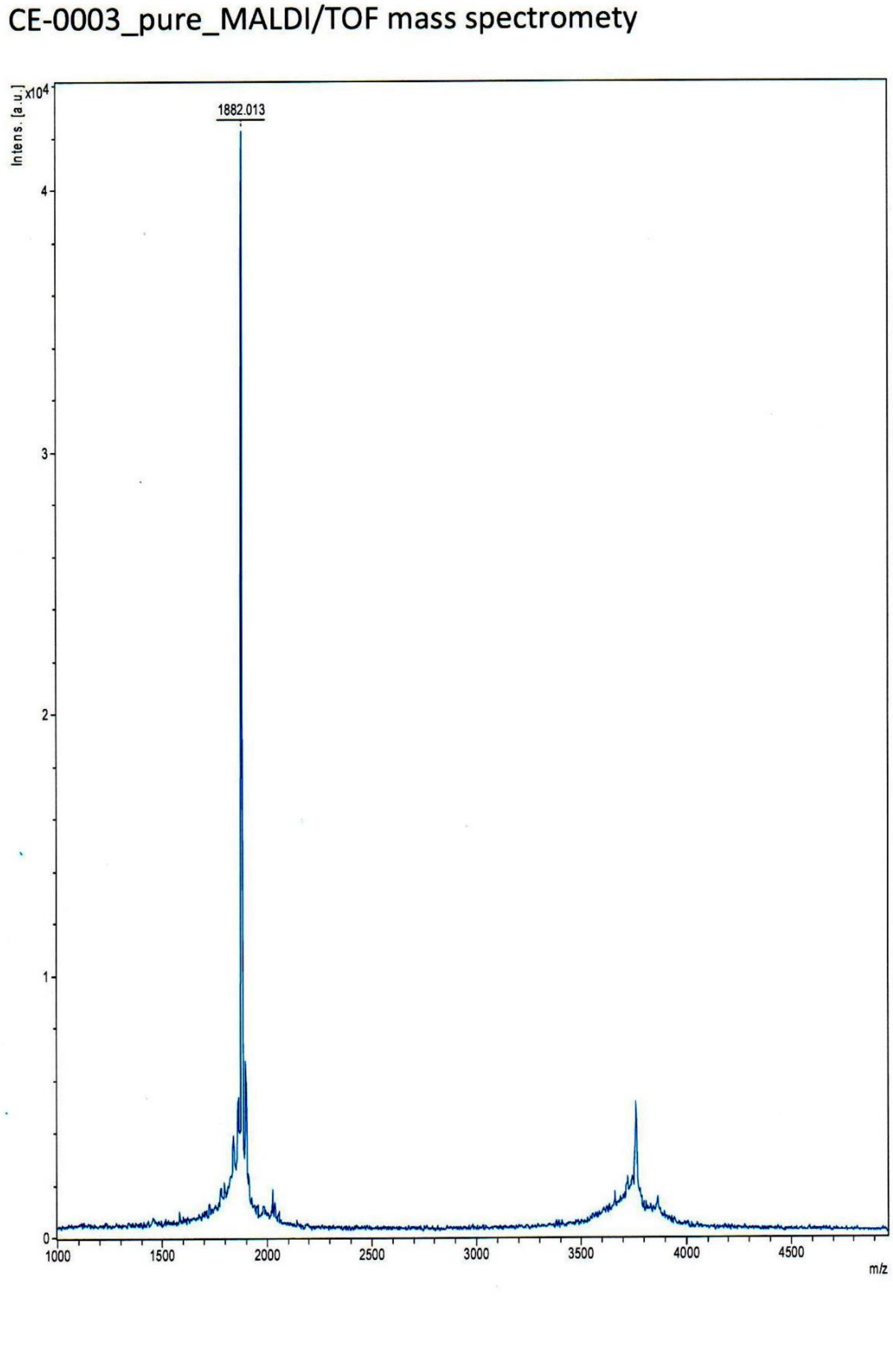

Figure S5. Mass spectrometry (MS) of CE-03.

SAMPLE INFORMATION

Sample Name:	cyclic-CE0005 Pk-2 Isomer pure	Acquired By:	System
Sample Type:	Unknown	Sample Set Name:	
Vial:	1	Acq. Method Set:	Analytical
Injection #:	1	Processing Method	Default
Injection Volume:	20.00 ul	Channel Name:	W2489 ChA
Run Time:	35.0 Minutes	Proc. Chnl. Descr.:	W2489 ChA 220nm
Date Acquired:	12/5/2022 2:33:15 PM EST		
Date Processed:	12/5/2022 3:19:55 PM EST		

Auto-Scaled Chromatogram

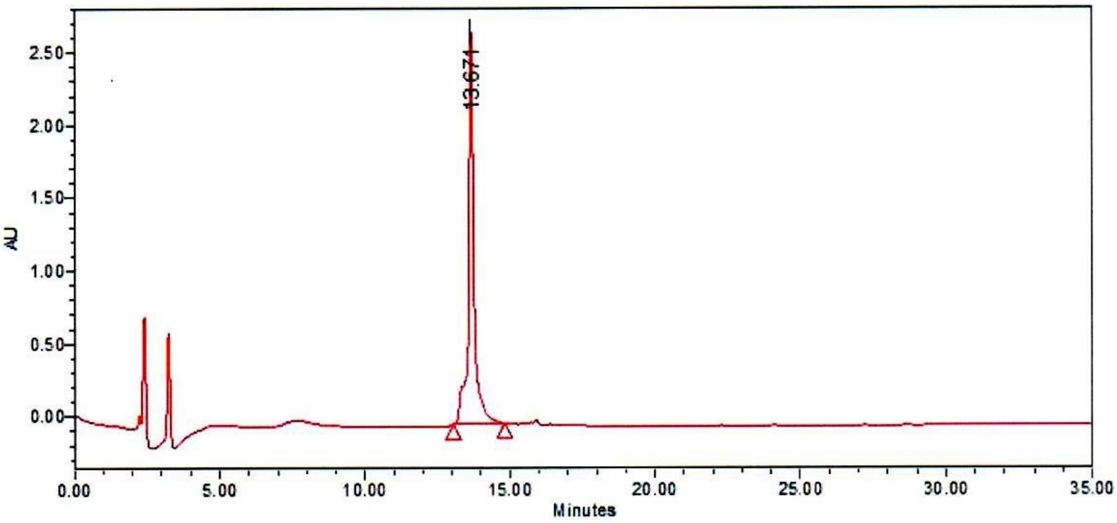

Column Information:

Waters C-4 Analytical column
3.5 micron 300A (250 x 4.6 mm)
UV Absorbance @ 220 nm
Injection 20ug in 50%TFE 0.1%TFA (aq.)
Mobile Phase : A: 0.1%TFA (aq.)
B: ACN 0.1%TFA

Gradient Conditions:

TIME	FLOW	%A	%B	CURVE
Initial	1.0	95	5	-
35	1.0	30	70	6
45	1.0	95	5	6
50	1.0	95	5	6
55	0	95	5	[illegible]

Peak Results

	Name	RT	Area	Height	Amount	Units
1		13.671	35724531	2715691		

Figure S6. High-pressure liquid chromatography (HPLC) of CE-05.

Cyclic-CE-0005_Pk-2 Isomer_pure_Bruker MALDI/TOF mass spectrometer

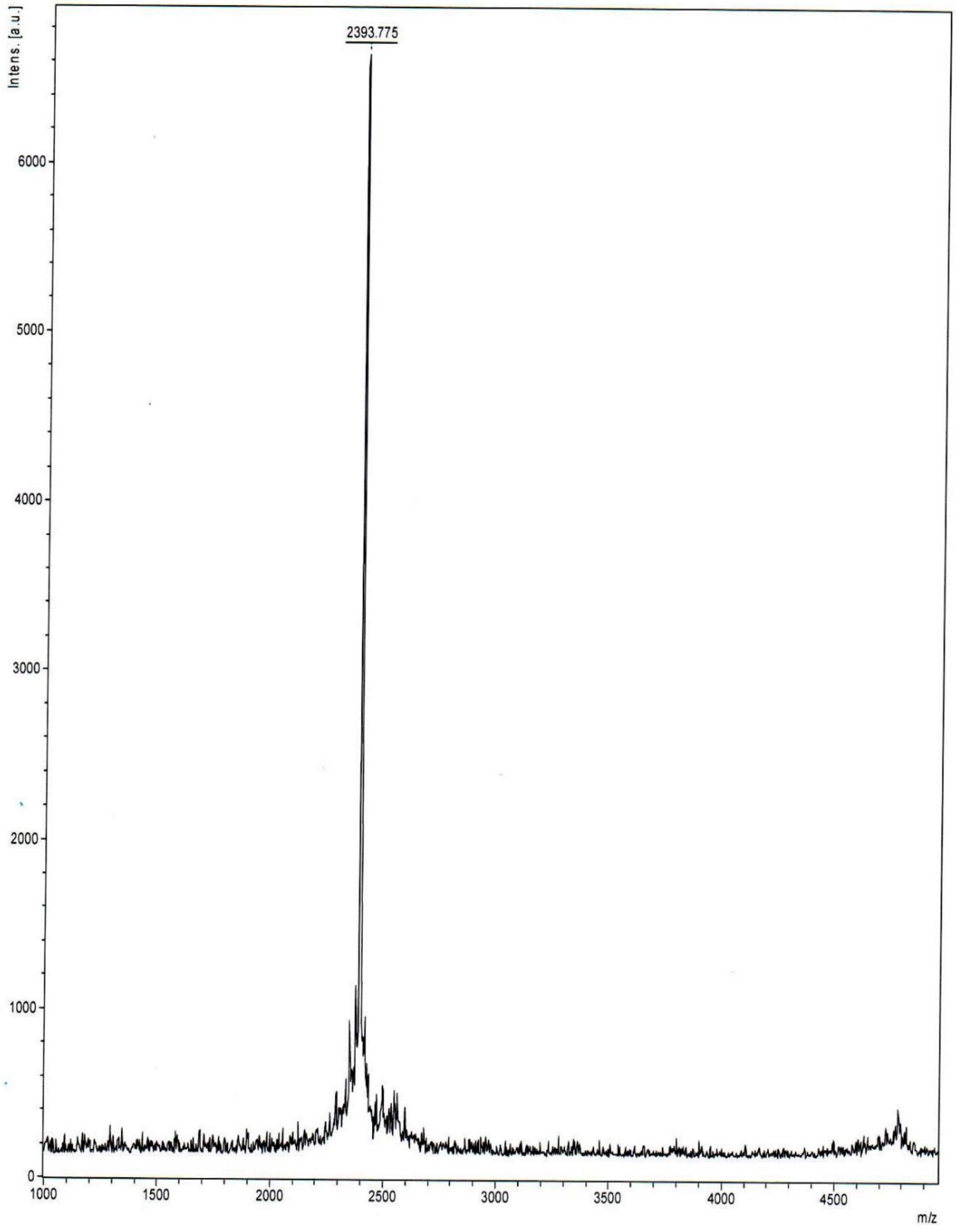

Figure S7. Mass spectrometry (MS) of CE-05.
